# A Case of Acquired Thrombotic Thrombocytopenic Purpura: Three Recurrences in 25 Years

**DOI:** 10.4274/tjh.2014.0361

**Published:** 2015-08-01

**Authors:** Şinasi Özsoylu

**Affiliations:** 1 Retired Professor of Pediatrics, Hematology and Hepatology, Honorary Fellow of American Academy of Pediatrics, Honorary Member of American Pediatric Society

**Keywords:** Thrombotic thrombocytopenic purpura

## TO THE EDITOR

A 60-year-old woman with fever, lassitude, neurological symptoms, hematuria, subconjunctival bleeding, several ecchymoses, reticulocytosis, microangiopathic hemolytic anemia (with burr cells, schistocytes, and spherocytes), and thrombocytopenia was first seen in the neurology clinic in 1985. Because of her normal cerebrospinal fluid and unremarkable MRI findings with some nonspecific EEG findings, thrombotic thrombocytopenic purpura (TTP) was diagnosed based on her clinical and microangiopathic hemolytic anemia findings, as reported previously. Her Coombs’ test and kidney and liver function results were almost normal and her moribund condition was treated with megadose methylprednisolone (MDMP) [1,2,3]. Her platelet count became normal on the fourth day of treatment; she did not require any blood transfusions and all clinical and laboratory findings were improved. Thereafter, she was seen once a year. Informed consent was obtained.

Fifteen years later, at the age of 75, her symptoms recurred with angiopathic findings without known infection, drug use, mild hematuria, or urinary system infection (E. coli above 100,000/mL). She was diagnosed with recurrence of TTP and was treated with antibiotics and oral MDMP (30 mg/kg for 3 days and then 20 mg/kg for 4 days; subsequently at doses of 10, 5, 2, and 1 mg/kg each for 1 week in duration), given once around 6 a.m. Her symptoms improved in terms of anemia and microangiopathic laboratory findings, including the thrombocytopenia, within a week without admission to the hospital. Her liver and kidney functions (urea: 30 mg/dL, creatinine: less than 1.1 mg/dL) stayed normal.

In 2007, 22 years after the first attack and at the age of 82, she had another episode of microangiopathic hemolytic anemia and thrombocytopenia without a known cause. She was being treated again with oral MDMP as of the year 2000. She responded in a 1-week period and stayed symptom-free for 3 years. Later it was learned that she had died at home at the age of 85 due to heart failure.

Thrombotic microangiopathic syndromes, though rare, have diverse pathophysiological pathways that can lead to microangiopathic anemia, a procoagulant state with or without damage to the kidneys and other organs, and could be acquired or hereditary [4,5].

Hereditary conditions related to hereditary ADAMTS 13 deficiency, complement H factor (CFH), complement 3-3b, complement C factor 1 (CF 1 or CD 45), cobalamin C disease, and some of the coagulation disorders such as thrombomodulin, plasminogen, and protein kinase C deficiencies could not be considered in this patient because of her age (first attack at about 60 years of age). Among the acquired disorders, Shiga toxin-mediated hemolytic and uremic syndrome (HUS), HELLP syndrome, drug immune-mediated conditions, and pregnancy-related microangiopathic syndromes could easily be rejected because of her age and no known drug history.

Although ADAMTS 13 deficiency related to antibodies was not studied (it was not known in 1985 when the patient was first seen), and permission for its determination could not be obtained for the patient’s second and third flare-ups, it was the most likely diagnosis for our patient at the time to our knowledge. MDMP was given intravenously during the first attack while she was in the hospital. On the second and third recurrences, it was administered orally at home.

This patient has been brought to our attention because of her prompt responses to MDMP in each recurrence and the absence of obvious organ damage, especially in terms of brain, liver, and kidney functions, until her death due to congestive heart failure at home. MDMP has been used for several immune-related disorders [6]. Since acquired TTP is most likely related to antibodies against ADAMTS 13, the response to MDMP in this disorder could easily be understood and should be studied in the future in more detail.

If other patients with acquired TTP with proven ADAMTS 13 deficiency respond to MDMP treatment without organ damage and early improvement of anemia and thrombocytopenia, this treatment could be the first choice for those patients.

**Conflict of Interest Statement**

The author of this paper has no conflict of interest, including specific financial interests, relationships, and/or affiliations relevant to the subject matter or materials included in this manuscript.
